# Shorter Telomeres in Peripheral Blood Mononuclear Cells from Older Persons with Sarcopenia: Results from an Exploratory Study

**DOI:** 10.3389/fnagi.2014.00233

**Published:** 2014-08-28

**Authors:** Emanuele Marzetti, Maria Lorenzi, Manuela Antocicco, Stefano Bonassi, Michela Celi, Simona Mastropaolo, Silvana Settanni, Vanessa Valdiglesias, Francesco Landi, Roberto Bernabei, Graziano Onder

**Affiliations:** ^1^Department of Geriatrics, Neurosciences and Orthopedics, Teaching Hospital “Agostino Gemelli”, Catholic University of the Sacred Heart School of Medicine, Rome, Italy; ^2^Unit of Clinical and Molecular Epidemiology, IRCCS San Raffaele Pisana, Rome, Italy; ^3^DICOMOSA Group, Department of Psychology, Area of Psychobiology, University of A Coruña, A Coruña, Spain

**Keywords:** frailty, biological age, muscle aging, oxidative stress, inflammation, bioelectrical impedance analysis

## Abstract

**Background:** Telomere shortening in peripheral blood mononuclear cells (PBMCs) has been associated with biological age and several chronic degenerative diseases. However, the relationship between telomere length and sarcopenia, a hallmark of the aging process, is unknown. The aim of the present study was therefore to determine whether PBMC telomeres obtained from sarcopenic older persons were shorter relative to non-sarcopenic peers. We further explored if PBMC telomere length was associated with frailty, a major clinical correlate of sarcopenia.

**Methods**: Analyses were conducted in 142 persons aged ≥65 years referred to a geriatric outpatient clinic (University Hospital). The presence of sarcopenia was established according to the European Working Group on Sarcopenia in Older People criteria, with bioelectrical impedance analysis used for muscle mass estimation. The frailty status was determined by both the Fried’s criteria (physical frailty, PF) and a modified Rockwood’s frailty index (FI). Telomere length was measured in PBMCs by quantitative real-time polymerase chain reaction according to the telomere/single-copy gene ratio (*T*/*S*) method.

**Results:** Among 142 outpatients (mean age 75.0 ± 6.5 years, 59.2% women), sarcopenia was diagnosed in 23 individuals (19.3%). The PF phenotype was detected in 74 participants (52.1%). The average FI score was 0.46 ± 0.17. PBMC telomeres were shorter in sarcopenic subjects (*T*/*S* = 0.21; 95% CI: 0.18–0.24) relative to non-sarcopenic individuals (*T*/*S* = 0.26; 95% CI: 0.24–0.28; *p* = 0.01), independent of age, gender, smoking habit, or comorbidity. No significant associations were determined between telomere length and either PF or the FI.

**Conclusion:** PBMC telomere length, expressed as *T*/*S* values, is shorter in older outpatients with sarcopenia. The cross-sectional assessment of PBMC telomere length is not sufficient at capturing the complex, multidimensional syndrome of frailty.

## Introduction

The remarkable inter-individual variability in functional and health status observed in late life indicates that chronological age *per se* does not precisely reflect the actual biological age of an organism (Mitnitski et al., 2002). This has instigated a great deal of research aimed at identifying clinical and biological parameters that are able to provide an overview of the health status, predict the risk of age-related diseases, and help estimate the remaining lifespan of an individual (Vasto et al., 2010).

Telomere biology has gained a special interest in the field of aging biomarkers (Lehmann et al., 2013). Telomeres are specialized structures located at the termini of mammalian chromosomes and consist of protein-bound, non-coding tandem-repeated hexamers (Blackburn, 1991). They serve to protect genome integrity by camouflaging chromosome ends from the DNA damage-response machinery, which would otherwise sense them as double-stranded breaks (Blackburn, 1991). In somatic cells, each round of DNA replication causes a loss of telomere repeats at the lagging strand, due to the presence of a terminal gap after degradation of the most distal primer. This phenomenon limits the total number of divisions normal somatic cells can undergo (Allsopp et al., 1992).

The observation that telomeres shorten over the life course and are implicated in cellular senescence has led to the hypothesis that telomere attrition may be a mechanism driving the aging process (Mikhelson and Gamaley, 2012). Indeed, associations have been determined between telomere erosion and premature aging syndromes, several age-sensitive measures (e.g., blood pressure, lung function, cognition, bone mineral density), age-related conditions (e.g., insulin resistance, type II diabetes mellitus, coronary artery disease, chronic obstructive pulmonary disease, dementia, cancer), and mortality [reviewed by Blasco (2005)]. As such, telomere length is considered to be an indicator of health status and, more in general, of biological age (Fossel, 2012).

At the clinical level, the assessment of muscle mass and function has emerged as a possible biomarker for aging (Fisher, 2004). Notably, the age-related loss in muscle mass and strength (sarcopenia) fulfills virtually all of the criteria defining an aging biomarker (Sprott, 2010). Indeed, sarcopenia (1) is an aging trait shared across species (Augustin and Partridge, 2009), (2) begins in adulthood and worsens over the course of aging (Frontera et al., 1991), (3) develops as a consequence of aging itself rather than being a mere correlate of other diseases (Iannuzzi-Sucich et al., 2002), (4) is not directly lethal, albeit impacting the health and functional status of an individual (Rolland et al., 2008), (5) is measurable and reproducible (Cruz-Jentoft et al., 2010), and (6) shows a clinical evolution that can be followed over relatively short periods of time (Marzetti, 2012).

The relationship between telomere attrition and muscle aging is currently unknown. The purpose of the present investigation was therefore to explore whether telomere length, measured in peripheral blood mononuclear cells (PBMCs), was associated with sarcopenia in a sample of older adults referred to a geriatric outpatient clinic. We further evaluated if PBMC telomere length was related to frailty, a major clinical consequence of sarcopenia (Roubenoff, 2000) and a possible additional clinical indicator of biological age (Mitnitski et al., 2002; Goggins et al., 2005).

## Materials and Methods

### Participant recruitment and setting

The study was conducted at the outpatient clinic of the Department of Geriatrics, Neurosciences and Orthopedics, Teaching Hospital “Agostino Gemelli,” Catholic University of the Sacred Heart (Rome, Italy). All patients aged 65+ years, admitted between October 2012 and January 2013, were invited to take part in the investigation. Exclusion criteria were: presence of disease conditions with an estimated life expectancy <6 months, inability to walk for 4 m, peripheral edema, presence of pacemaker or implantable cardioverter defibrillator, and unwillingness or inability to provide informed consent. The study was approved by the Institutional Review Board of the Catholic University of the Sacred Heart, and all participants signed a written consent. Study visits for physical function testing, body composition assessment, and blood sampling were scheduled within a week of enrollment.

### Data collection

Demographic, clinical data, and lifestyle habits were collected at the time of enrollment through a dedicated questionnaire. Disability status was evaluated by the Katz’s Activities of the Daily Living (ADL) (Katz and Akpom, 1976). Cognition was assessed using the mini-mental state examination (MMSE) (Folstein et al., 1975), while mood was evaluated by the 15-item Geriatric Depression Scale (GDS) (Sheikh and Yesavage, 1986). Diagnoses were gathered from the patient, attending physicians, and the careful review of medical charts. Finally, the comorbidity burden was calculated via the Cumulative Illness Rating Scale (CIRS) (Linn et al., 1968).

### Identification of sarcopenia

The presence of sarcopenia was established according to the European Working Group on Sarcopenia in Older People (EWGSOP) criteria (Cruz-Jentoft et al., 2010). Whole-body fat-free mass was measured by bioelectrical impedance analysis (BIA) using a Quantum/S Bioelectrical Body Composition Analyzer (Akern Srl, Florence, Italy) with an operating frequency of 50 kHz at 800 μA. Measurements were taken under standard conditions, with the subject in a supine position and surface electrodes placed on the right wrist and ankle (NIH Expert Panel, 1996). Muscle mass was estimated using the equation developed by Janssen et al. (2000). The skeletal muscle index [SMI (kg/m^2^)] was obtained dividing absolute muscle mass by squared height. According to the EWGSOP indications, low SMI was defined based on the following cut-offs: <8.87 kg/m^2^ in men and <6.42 kg/m^2^ in women (Cruz-Jentoft et al., 2010).

### Definition of frailty status

The frailty status of participants was assessed according to both the Fried’s criteria (physical frailty, PF) (Fried et al., 2001) and a modified Rockwood’s frailty index (FI) (Searle et al., 2008). The following indicators were considered to define PF: (1) unintentional weight loss in prior 12 months; (2) poor endurance and energy; (3) weakness, defined by poor grip strength; (4) slowness, assessed via timed 4-m walk speed; and (5) low physical activity level according to the Physical Activity Scale for the Elderly (PASE) (Washburn et al., 1993) (Table 1).

**Table 1:** **Indicators of physical frailty**.

Frailty criteria	Parameters
Weight loss	Loss of ≥5 kg in prior 12 months, unintentional
Exhaustion	Response of “a moderate amount of the time (3–4 days)” or “most of the time” to the CES-D scale item: “I felt that everything I did was an effort” during the past week
Weakness	Low grip strength assessed by a North Coast handheld dynamometer. Gender- and BMI-specific cutoff points provided by Fried et al. (2001) were adopted
Slowness	Time in seconds to complete a 4-m walk at usual pace. Gender- and height-specific cutoff points provided by Fried et al. (2001) were adopted
Low physical activity levels	Physical Activity Scale for the Elderly (PASE); cut-points: Men < 64, women < 52 (Rothman et al., 2008)

**Frailty status**	**Number of criteria**

Robust	0
Pre-frail	1–2
Frail	≥3

*BMI, body mass index; CES-D, Center for Epidemiologic Studies-Depression*.

The participant frailty status was further evaluated by constructing a FI based on the summation of “health deficits” principle (Rockwood et al., 2005), according to the procedure described by Searle et al. (2008). A total of 30 deficits were used for the construction of the FI, which is expressed as the ratio of deficits observed to the total number of deficits considered. The variables used for the computation of the FI and their corresponding cut-points are listed in Table 2.

**Table 2:** **Health variables and cut-points used for the computation of a modified Rockwood’s frailty index**.

Health variables	Condition or variable cut-points	Score
Marital status	Married or single	0
	Widow or divorced	1
Social involvement	Yes	0
	No	1
Impaired ADL	None	0
	1	0.25
	2	0.5
	3–4	0.75
	4–6	1
Impaired IADL	None	0
	1–2	0.25
	3–4	0.5
	5–6	0.75
	7–8	1
Walk speed (4 m)	≥0.8	0
	<0.8	1
Grip strength^a^	Normal	0
	Low	1
Cognition (MMSE)	>24	0
	20–24	0.25
	18–20	0.50
	11–17	0.75
	<10	1
Mood (GDS)	0–2	0
	3–5	0.25
	6–8	0.5
	9–11	0.75
	>11	1
Sedentarism (PASE)	Men ≥ 64, women ≥ 52	0
	Men < 64, women < 52	1
Hospital admission(s) in prior 12 months	No	0
	Yes	1
BMI	18.5–24.9	0
	25–30	0.5
	>30 or <18.5	1
Nutrition (MNA)	≥23.5	0
	≥17 < 23.5	0.5
	<17	1
Unintentional weight loss (>5 kg in prior 12 months)	No	0
	Yes	1
Chronic pain	No	0
	Yes	1
Fall(s) in prior 12 months	No	0
	Yes	1
Cancer or active cancer treatment	No	0
	Yes	1
Cardiovascular disease	No	0
	Yes	1
Chronic lung diseases	No	0
	Yes	1
Hematological diseases	No	0
	Yes	1
Renal diseases	No	0
	Yes	1
Central nervous system diseases	No	0
	Yes	1
Peripheral nervous system diseases	No	0
	Yes	1
Gastrointestinal diseases	No	0
	Yes	1
Ear, nose, and throat diseases	No	0
	Yes	1
Orthopedic diseases	No	0
	Yes	1
Psychiatric disorders	No	0
	Yes	1
Diabetes mellitus	No	0
	Yes	1
Endocrine disorders	No	0
	Yes	1
Sarcopenia	No	0
	Yes	1
Polypharmacy (≥6 drugs)	No	0
	Yes	1

*^a^Men: 1, BMI ≤ 24, grip strength ≤ 29 kg; BMI 24.1–28, grip strength ≤30 kg; BMI > 28, grip strength ≤ 32 kg. Women: 1, BMI ≤ 23, grip strength ≤ 17 kg; BMI 23.1–26, grip strength ≤ 17.3 kg; BMI 26.1–29, grip strength ≤ 18 kg; BMI > 29, grip strength ≤ 21 kg*.

*ADL, activities of daily living; BMI, body mass index; IADL, instrumental activities of daily living; GDS, Geriatric Depression Scale; MMSE, mini-mental state examination; MNA, Mini nutritional assessment; PASE, physical activity scale for the elderly*.

### Blood sampling and processing

Blood samples were obtained by Vacutainer™ venipuncture of the median cubital vein after overnight fasting. Blood samples (10 mL) were diluted 1:1 in phosphate-buffered saline (PBS) and PBMCs separated within 1 h of blood draw by Ficoll-Hypaque (Comercial RAFER, Zaragoza, Spain) density gradient following the manufacturer’s instructions. Cells were washed twice with PBS and cryopreserved at -80°C in RPMI 1640 containing 50% fetal bovine serum and 10% dimethylsulfoxide.

### Measurement of telomere length by quantitative real-time polymerase chain reaction

Genomic DNA was extracted from isolated PBMCs using a commercial DNA isolation kit (Norgen Biotek, Thorold, Canada) as per the manufacturer’s instructions. Relative telomere length was measured by quantitative real-time polymerase chain reaction (qRT-PCR) according to the telomere/single-copy gene ratio (*T*/*S*) method (Cawthon, 2002) with minor modifications. Briefly, the method measures the ratio between the copy number of telomere repeats (*T*) and that of the single-copy gene 36B4 (*S*) used as a quantitative control, relative to a calibrator sample (human genomic DNA; Roche Diagnostic, Indianapolis, IN). qRT-PCR was performed using an Applied Biosystems 7300 RT-PCR System (ABI, Foster City, CA) with the following cycling conditions: 95°C for 10 min, 40 cycles at 95°C for 5 s, 56°C for 30 s, 72°C for 30 s. *T* and *S* were analyzed in duplicate within the same plate. The same calibrator sample was included in all plates to allow comparisons across runs. A no-template control was also included for quality control. The relative *T*/*S* values were calculated according to the 2^-ΔΔCT^ method (Livak and Schmittgen, 2001).

### Statistical analyses

All data are expressed as proportions (%) or mean ± SD. Given the non-normal distribution of *T*/*S* values, analyses were run using log-transformed values to ensure equality of variances and render the errors approximately normally distributed. Analysis of covariance (ANCOVA) was used to compare adjusted means of log *T*/*S* values according to sarcopenia, SMI categories, and frailty. Geometric means of *T*/*S* values are shown in tables and text. Analyses were adjusted for age, gender, smoking habit, presence of diabetes, and CIRS score. The Spearman’s rank correlation coefficient was used to calculate the strength of association between variables. All analyses were performed using the SPSS software (version 18, SPSS Inc., Chicago, IL, USA).

## Results

A total of 142 subjects were enrolled in the study. The main characteristics of the study sample are shown in Table 3. Sarcopenia was identified in 23 participants (19.3%). The prevalence of sarcopenia was uniform across ages and genders. Frailty, as determined by the Fried’s criteria (PF), was detected in 74 participants (52.1%). Individuals classified as frail according to PF were older relative to non-frail subjects (76.6 ± 6.7 vs. 73.1 ± 6.0 years; *p* = 0.001), with no differences between genders. An identical prevalence of frailty was observed using 0.44 as the cutoff for the FI, as recommended by Rockwood et al. (2007). Similar to PF, participants with a FI score ≥0.44 were older than those with lower scores (76.4 ± 6.6 vs. 73.2 ± 6.2 years; *p* = 0.004), with an equal gender distribution. Sixty-one participants were identified as frail based on both PF and the FI score, and the two measures of frailty were significantly correlated with each other (*r* = 0.63; *p* < 0.0001). The strength of this correlation is comparable to that reported using the original version of the FI (Rockwood et al., 2007). Hence, the modified FI constructed for the present study was able to capture the condition of interest. The coexistence of sarcopenia and PF was observed in 21 subjects (91.3%), whereas 15 (65.2%) participants with sarcopenia were classified as frail according to the FI.

**Table 3:** **Study sample characteristics**.

	Whole sample (*n* = 142)
	*n* (%)
Age, years (mean ± SD)	74.9 ± 6.5
Female gender	84 (59.2)
Smokers	11 (7.7)
Education, years (mean ± SD)	10.0 ± 5.0
Hospital admission in prior 12 months	41 (29.0)
MMSE score (mean ± SD)	26.2 ± 3.4
CIRS (mean ± SD)	3.0 ± 2.2
GDS (mean ± SD)	10.8 ± 7.6
ADL scale (mean ± SD)	5.0 ± 1.3
IADL scale (mean ± SD)	5.9 ± 2.4
Fall in prior 12 months	66 (46.5)
BMI (mean ± SD)	27.7 ± 4.7
Number of drugs (mean ± SD)	6.1 ± 3.3
Frail (Fried’s criteria, PF)	74 (52.1)
Number of frailty criteria (mean ± SD)	2.3 ± 1.8
Frail (modified Rockwood’s frailty index, FI)	74 (52.1)
FI (mean ± SD)	0.46 ± 0.17
Sarcopenia (EWGSOP criteria)	23 (19.3)
PBMC telomere length (*T*/*S*)	0.27 ± 0.10

*ADL, activities of daily living; BMI, body mass index; CIRS, Cumulative Illness Rating Scale; EWGSOP, European Working Group on Sarcopenia in Older People; FI, frailty index; GDS, Geriatric Depression Scale; IADL, instrumental activities of daily living; MMSE, mini-mental state examination; PBMC, peripheral blood mononuclear cell; PF, physical frailty*.

**Figure 1:**
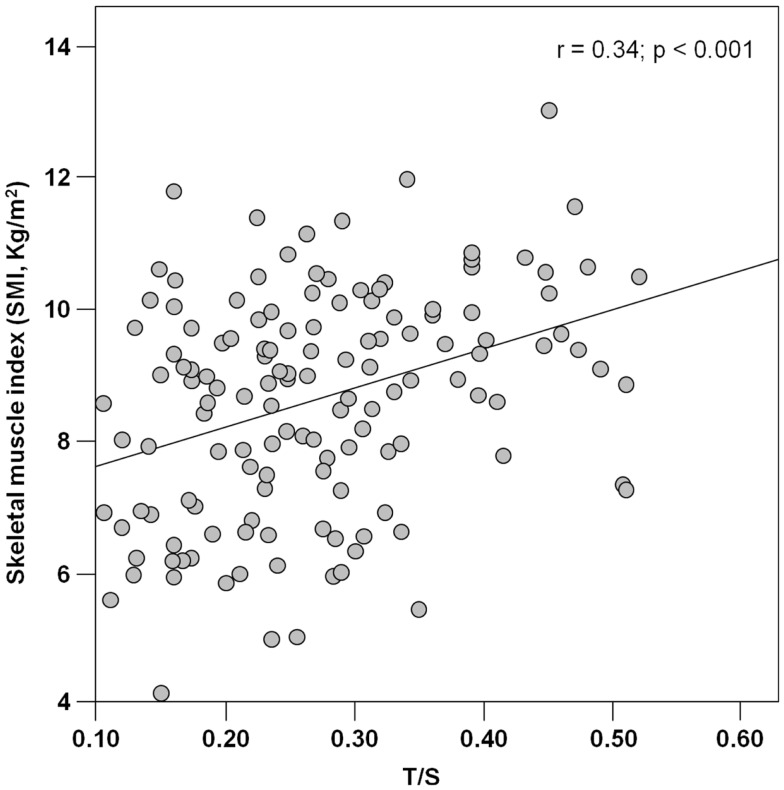
**Scatter plot of telomere/single-copy gene ratio (*T/S*) and the skeletal muscle index (*n* = 142)**.

PBMC telomeres were shorter in sarcopenic subjects (*T*/*S* = 0.21; 95% CI: 0.18–0.24) relative to non-sarcopenic individuals (*T*/*S* = 0.26; 95%: CI: 0.24–0.28; *p* = 0.01), independent of age, gender, smoking habit, presence of diabetes, and comorbidity (Table 4). Of the three parameters considered for the definition of sarcopenia (i.e., muscle mass, gait speed, and handgrip strength), *T*/*S* was only correlated with SMI (Figure 1).

**Table 4:** **Mean telomere/single-copy gene ratio (*T*/*S*) values according to sarcopenia, skeletal muscle index, and frailty status**.

	Mean telomere/single-copy gene ratio (*T*/*S*)			
	Unadjusted	*p*	Adjusted^b^	*p*
	mean^a^ (95% CI)		mean^a^ (95% CI)	
**SARCOPENIA (EWGSOP DEFINITION)**				
No sarcopenia (*n* = 119)	0.26 (0.25–0.28)	0.004	0.26 (0.24–0.28)	0.01
Sarcopenia (*n* = 23)	0.21 (0.17–0.24)		0.21 (0.18–0.24)	
**SKELETAL MUSCLE INDEX (SMI)**				
Normal SMI (*n* = 116)	0.26 (0.24–0.28)	0.003	0.26 (0.24–0.28)	0.008
Low SMI (*n* = 26)	0.21 (0.17–0.24)		0.21 (0.18–0.24)	
**FRAILTY, PF**				
No frailty (*n* = 68)	0.27 (0.24–0.29)	0.11	0.26 (0.24–0.29)	0.31
Frailty (*n* = 74)	0.24 (0.22–0.26)		0.24 (0.22–0.27)	
**FRAILTY, FI ≥ 0.44**				
No frailty (*n* = 68)	0.27 (0.24–0.29)	0.12	0.26 (0.24–0.29)	0.38
Frailty (*n* = 74)	0.23 (0.22–0.26)		0.24 (0.22–0.27)	

*Low SMI was defined as SMI < 8.87 kg/m^2^ for men and SMI < 6.42 kg/m^2^ for women*.

*^a^Geometric means were calculated from log-transformed values*.

*^b^Adjusted for age, gender, smoking habit, diabetes, and Cumulative Illness Rating Scale score*.

PBMC telomeres showed a trend toward lower *T/S* values in frail subjects relative non-frail participants, but the difference did not reach the statistical significance in either unadjusted or adjusted analyses, regardless of the frailty assessment tool adopted (Table 4). Finally, no significant correlations were determined between *T/S* values and any of the five domains defining PF (data not shown).

## Discussion

Previous studies have shown that telomere attrition and dysfunction are implicated in a host of age-related disorders, including cancer, cardiovascular disease, type 2 diabetes mellitus, osteoarthritis, chronic obstructive pulmonary disease, dementia, and immunosenescence [reviewed by Xi et al. (2013)]. However, the literature is void of investigations concerning the relationship between telomere length and sarcopenia, a hallmark of the aging process (Fisher, 2004). Furthermore, only sparse reports exist that have examined the association between telomere length and frailty (Woo et al., 2008; Collerton et al., 2012), an additional clinical indicator of biological age (Mitnitski et al., 2002; Goggins et al., 2005) and a major consequence of sarcopenia (Roubenoff, 2000). The present investigation was therefore undertaken to explore whether a popular senescence biomarker (PBMC telomere length) was related to clinical measures of biological age (sarcopenia and frailty). Our results indicate that PBMC telomere length, expressed as *T/S* values, is associated with sarcopenia, but not frailty, in a sample of older outpatients.

These findings are supportive of the proposition that sarcopenia may serve as a clinical biomarker for aging (Fisher, 2004). The inverse association detected between PBMC telomere length and muscle mass could be reflective of a common pathogenic ground underlying age-related telomere shortening and muscle atrophy. Indeed, oxidative stress and chronic inflammation are involved both in telomere erosion (Aviv, 2004; Bayne and Liu, 2005) and sarcopenia (Marzetti et al., 2013). The exposure to high levels of free radicals has been identified as a causative factor for telomere shortening both *in vitro* (Richter and von Zglinicki, 2007) and in disease conditions characterized by enhanced oxidant generation, such as diabetes mellitus, dementia, cardiovascular disease, and cancer [reviewed by Aubert and Lansdorp (2008)]. Likewise, oxidative stress is a major culprit in the development of sarcopenia through irreversible damage to myocyte macromolecules, bioenergetic failure, and induction of apoptosis [reviewed by Marzetti et al. (2009) and Calvani et al. (2013)].

A major consequence of oxidative stress is the activation of redox-sensitive mediators, including nuclear factor-κB (NF-κB) (Chung et al., 2009). The latter, in turn, regulates the transcription of several pro-inflammatory cytokines (Chung et al., 2009). Under normal conditions, NF-κB activation in response to oxidative stimuli is short-lived, and the inflammatory reaction ceases with resolution. However, the long-term exposure to high levels of oxidants, as it seems to occur during aging, results in a chronic activation of NF-κB-mediated inflammatory response and cellular damage (Chung et al., 2006). Notably, increases in circulating levels of C-reactive protein (CRP) and serum amyloid A (SAA) were associated with proportional decreases in PBMC telomere length in a cohort of middle-aged workers exposed to occupational environmental pollution over 2 years of follow-up (Wong et al., 2014). In addition, cross-sectional analyses in a population of 1,962 older adults ranging in age between 70 and 79 years showed that individuals with elevated circulating levels of either interleukin-6 (IL-6) or tumor necrosis factor-alpha (TNF-α) had significantly higher odds for short PBMC telomeres, after adjustment for potential confounders (O’Donovan et al., 2011). Remarkably, the highest odds for short PBMC telomeres were found in older persons with high levels of both IL-6 and TNF-α (O’Donovan et al., 2011).

It is noteworthy that IL-6 (Payette et al., 2003), TNF-α (Pedersen et al., 2003), CRP (Cesari et al., 2005), and SAA (Zhang et al., 2009) have all been implicated in the pathogenesis of muscle atrophy in the context of sarcopenia or other muscle-wasting disorders. Similar to telomere attrition, the concomitant elevation of multiple inflammatory markers seems to play a synergistic role in age-related muscle loss (Visser et al., 2002).

The association between telomere length and sarcopenia was mainly driven by the relationship between *T/S* values and muscle mass. Indeed, of the three parameters indicated by the EWGSOP for the definition of sarcopenia (Cruz-Jentoft et al., 2010), PBMC telomere length was only correlated with SMI (Figure 1). The reasons for the absence of significant associations between *T/S* values and measures of muscle performance (handgrip strength and walk speed) are multifold. First, it is well known that losses in muscle mass and function follow different temporal trajectories during the course of aging, with steeper declines in strength relative to mass (Delmonico et al., 2009). Hence, at any given time point, PBMC telomere length may not necessarily correlate with all of the components of the sarcopenia syndrome. In addition, while SMI is intrinsic to muscle, force generation and ambulation depend on the coordinated function of multiple organ systems (i.e., musculoskeletal, cardiorespiratory, and central and peripheral nervous systems). Since the rate of aging varies across organs and tissues (Finkel et al., 1995), a single biological marker may not be equally effective at tracking the multitude of intrinsic and extrinsic factors responsible for muscle aging. A similar reasoning may explain the inability of PBMC telomere length measurements to capture the complex inter-organ interactions regulating muscle performance.

Since frailty has been proposed as a clinical meter for biological age (Mitnitski et al., 2002; Goggins et al., 2005) and represents a major consequence of sarcopenia (Roubenoff, 2000), one could have expected a relationship existed between PBMC telomere length and the frailty status. However, the lack of a significant association between *T/S* values and measures of frailty is in keeping with previous reports on the topic (Woo et al., 2008; Collerton et al., 2012). Similar to the present study, PBMC telomere length was indeed unrelated to either PF (Collerton et al., 2012) or the FI (Woo et al., 2008; Collerton et al., 2012). As previously reasoned with regard to muscle function, it is conceivable that the “snapshot” assessment of a single biological marker may not be sufficient at capturing a complex, multidimensional syndrome, such as frailty.

Although reporting novel findings, the present work presents some limitations that deserve further discussion. First of all, the study is exploratory in nature, evident by the relatively small sample size. For this reason, robust and pre-frail participants were considered as a single group, which prevented us from observing a possible gradient of *T/S* values across the frailty spectrum. Nevertheless, this approach allowed adjusting the analyses for a number of potential confounders, which adds further relevance to our findings. Second, the cross-sectional design of the study does not allow inferring about the temporal relationship among PBMC telomere length, frailty, and sarcopenia. Moreover, although BIA is an established technique for the estimation of lean body mass (Kyle et al., 2003), it does not represent the gold standard for the quantification of muscle mass. Nevertheless, BIA is safe, inexpensive, easy to use, and readily reproducible. This technique is indeed recommended by the EWGSOP for the estimation of muscle mass in ambulatory patients (Cruz-Jentoft et al., 2010), such as those enrolled in the present study. Furthermore, following the recommendations by the NIH Expert Panel (1996), BIA measurements were obtained under standard conditions to limit the possible variability arising from body position, hydration status, consumption of food and beverages, ambient air and skin temperature, recent physical activity, and conductance of the examining table. Finally, telomere length was estimated from *T/S* values, as determined by qRT-PCR, in place of absolute quantification by classic Southern blot methods on terminal restriction fragments. However, the *T/S* method has proven to be highly consistent with Southern blot (Epel et al., 2004; Grabowski et al., 2005).

## Conclusion

Findings from this exploratory study indicate that PBMC telomeres are shorter in sarcopenic geriatric outpatients, after adjustment for potential confounders. The relationship between telomere length and sarcopenia appears to be mainly driven by muscle mass, which may be indicative of a common pathogenic ground for telomere erosion and muscle atrophy. The lack of a significant association between PBMC telomere length and measures of muscle performance or the frailty status reinforces the notion that telomere shortening may not suffice as a biomarker for complex, multidimensional age-related conditions (Woo et al., 2008; Collerton et al., 2012). Future studies are necessary to assess the relationship among telomere shortening, sarcopenia, and frailty over time as well as in response to interventions, such as physical exercise and nutrition, proven effective against muscle aging and its clinical correlates.

## Conflict of Interest Statement

The authors declare that the research was conducted in the absence of any commercial or financial relationships that could be construed as a potential conflict of interest.
